# Epidemiological patterns and healthcare utilization among malaria patients in Sorong City, Papua, Indonesia: A cross-sectional analysis of national surveillance data

**DOI:** 10.1371/journal.pone.0350434

**Published:** 2026-06-04

**Authors:** Tri Nugraha Susilawati, Herry Susanto, Oanh Kieu Nguyet Pham, Federika Kondororik, Vivian Nanny Lia Dewi, Hamdiah Ahmar, Noviyati Rahardjo Putri, Rogan Lee, Minh Cuong Duong

**Affiliations:** 1 Faculty of Medicine, Universitas Sebelas Maret, Surakarta, Indonesia; 2 Faculty of Nursing, Universitas Islam Sultan Agung, Semarang, Indonesia; 3 Hospital for Tropical Diseases, Ho Chi Minh City, Vietnam; 4 Department of Health, Population Control and Family Planning – Ministry of Health, Sorong, Indonesia; 5 Goods and Services Procurement Bureau – Regional Secretariat of Southwest Papua Province, Sorong,‌‌ Indonesia; 6 Faculty of Health, Universitas Aisyah Pringsewu, Lampung, Indonesia; 7 Faculty of Medicine, Universitas Papua, ‌‌Sorong Regency, Indonesia; 8 Centre for Infectious Diseases and Microbiology Laboratory Services, Institute of Clinical Pathology and Medical Research – New South Wales Health Pathology, Westmead Hospital, Westmead, Australia; 9 School of Medical Sciences, Faculty of Medicine and Health, The University of Sydney, Westmead Hospital, Westmead, Australia; 10 School of Population Health, University of New South Wales, Sydney, Australia; Menzies School of Health Research, AUSTRALIA

## Abstract

**Objectives:**

Malaria remains endemic in Indonesia, with high transmission rates observed in the Papua region. Routine surveillance data are essential to inform service delivery and optimize case management. This cross-sectional study examined malaria cases in Sorong City in 2024 reported through Indonesia’s national surveillance system to describe malaria burden across healthcare facilities, diagnostic and treatment practices, and predictors of hospital attendance and hospitalization.

**Methods:**

All laboratory-confirmed malaria cases were analyzed, including demographics, malaria diagnosis, disease severity, treatment received, and hospitalization data. Descriptive statistics and logistic regression were used to identify determinants of hospital attendance and inpatient admission.

**Results:**

Among 3,953 malaria cases, most patients were ≥15 years old (65.9%), males (59.4%), Papuan (54.2%), students (36.6%), and living in highly endemic areas (78%). Community health centers reported most cases (61.7%). Microscopy was the primary diagnostic tool (70.1%). *Plasmodium vivax* was the predominant species (65.5%), and nearly all infections were uncomplicated (99.9%). Notably, nearly 21% of patients received non-standard antimalarial regimens. Predictors of hospital attendance include older age (aOR: 1.02; 95% CI: 1.00–1.02; p = 0.001), non-Papuans ethnicity (aOR: 1.77; 95%CI: 1.51–2.07; p = 0.001), being housewife (aOR: 1.57; 95%CI: 1.02–2.42; p = 0.041) or student (aOR: 1.56; 95%CI: 1.05–2.31; p = 0.029), and living in moderate- (aOR: 2.01; 95%CI: 1.69–2.40; p = 0.001) or low- (aOR: 4.57; 95%CI: 2.01–10.39; p = 0.001) endemicity areas. Children <15 years were more likely to be hospitalized (aOR 1.9; 95% CI 1.18–3.06; p = 0.009).

**Conclusions:**

Malaria remains a substantial public health burden, dominated by *P. vivax*, with community health centers serving as primary care providers. Older individuals were more likely to attend hospital, while younger children had a higher likelihood of hospitalization once diagnosed. Strengthening community-based services, promoting early treatment-seeking among children, and ensuring consistent adherence to national treatment guidelines are critical to reducing severe disease and hospital burden.

## Introduction

Malaria remains a major global public health challenge, with an estimated 282 million cases and 610,000 deaths globally in 2024 [1]. Indonesia is the second-largest contributor to malaria cases in the WHO Western Pacific region, accounting for 25% of all regional cases [1]. Indonesia recorded 543,965 cases in 2024, with Papua contributes more than 93% of national malaria cases despite comprising only a small proportion of the national population [2,3]. This figure indicates a 30% increase in malaria cases in Indonesia compared to 2023, despite the launch of a malaria elimination program in 2009 with a target of elimination by 2030 [3,4]. To achieve the district and city target of malaria elimination, several activities are being carried out including improving the quality of malaria diagnostics, the use of artemisinin-based combination therapy (ACT), malaria surveillance, and vector control [5]. However, the percentage of positive malaria patients who received appropriate treatment may not have reached the target because the budget was unavailable from the beginning of the year, resulting in a stockout of antimalarial drugs [6].

Despite substantial progress in some regions, Papua continues to experience persistent transmission [7]. Control efforts are complicated by the presence of multiple Plasmodium species; i.e., *P. falciparum*, *P. vivax*, *P. malariae*, and *P. ovale* [8]. Because species determination in routine surveillance often relies on microscopy and/or RDTs, misclassification can occur and rare-species reports should be interpreted cautiously [9,10]. Importantly, the predominance of *P. vivax* in many Papuan settings poses additional operational challenges due to relapse risk and the need for effective radical cure and adherence to primaquine regimens [8].

Indonesia’s malaria treatment guidelines recommend dihydroartemisinin–piperaquine (DHP) for three days plus primaquine for uncomplicated malaria, with species-specific dosing regimens [11]. Uncomplicated *P. falciparum* is treated with DHP and a single, low-dose (0.25 mg/kg on day 1) primaquine, while *P. vivax* and *P. ovale* require a 14-day primaquine regimen for radical cure. Relapsing *P. vivax* infections require higher primaquine dosing (0.5 mg/kg/day for 14 days) after G6PD testing. Severe malaria is treated with intravenous artesunate until the patient can take oral medication. The use of primaquine is avoided in pregnancy and infancy.

Sorong City in Southwest Papua (previously West Papua) province, remains a high-incidence setting. The annual parasite incidence (API) in the province is 7.56 in 2021 and increased to 12.68 in 2022 and 12.47 in 2023 [12–14]. There was an increase of *P. vivax* infection in this area, replacing the previously high burden of *P. falciparum*. Falciparum malaria contributed to 48.56% of total cases in Sorong City in 2021, then declined to 41.65% in 2022 and 36.81% in 2023. In contrast, *P. vivax* infection increased from 39.58% in 2021 to 44.91% in 2022 and 58.16% in 2023 [15]. Patients often experience mild symptoms and preferentially seek care at community health centers (CHCs) [16]. However, limited evidence exists on the factors influencing health-facility attendance (e.g., primary care vs hospital) or predictors of hospitalization using routine surveillance data, particularly in settings where cases may be detected through both passive case detection (PCD) and CHC-led active case detection (ACD). Understanding pathways to care, clinical management, and the current malaria burden, including associated risk factors in these settings is essential for informing malaria control and elimination strategies. This study therefore aimed to analyze national surveillance data from Sorong City to: (1) assess malaria prevalence and severity across healthcare facilities, (2) describe diagnostic and treatment practices, and (3) identify predictors of hospital attendance and hospitalization among malaria patients.

## Methods

### Study design, context, and source of data

A cross-sectional study was conducted to examine malaria cases in Sorong City, Southwest Papua Province, Indonesia, between 1 January and 31 December 2024. Data were obtained from SISMAL (Sistem Informasi Surveilans Malaria), the national malaria surveillance system developed by the Indonesian Ministry of Health to collect cases reported from all 38 provinces across the country (https://sismal.kemkes.go.id/). All healthcare facilities in Sorong City report their malaria cases to SISMAL, allowing case monitoring at national, provincial, and city levels. Patients’ data, including demographic information, clinical information, laboratory test results, and treatments are documented in both medical records and the SISMAL database. It is important to note that SISMAL contains mandatory and non-mandatory inputs. To maintain data integrity, patient-reported clinical symptoms were excluded from the analysis due to the non-mandatory nature of these fields in SISMAL, which resulted substantial missingness and a high risk of reporting bias.

At each healthcare facility, the malaria program manager is responsible for entering case data into SISMAL for routine monitoring. Information is extracted from medical and laboratory records, and the system is periodically synchronized with the national server [13]. However, based on our observation, there is no routine, independent cross-verification of individual records at the point of data entry. Consequently, data quality and reliability largely depend on the accuracy of source documentation and the diligence of the personnel responsible for data entry, and some apparent “errors” may reflect electronic reporting/data entry issues rather than clinical practice. In Sorong City, malaria patients can seek treatment at three different types of health facilities, including clinics, CHCs, or hospitals. At CHCs, health workers routinely conduct active case finding (ACD) through home visits and screening high-risk groups in addition to treat those who present for care (PCD), while clinics and hospitals only report malaria cases who visit these facilities (PCD). RDTs are commonly used for ACD during outreach activities and may also be used for PCD when microscopy is not available (e.g., after-hours service or operational constraints); this reason is not systematically recorded in SISMAL. Anonymous data from SISMAL were requested from the Ministry of Health of the Republic of Indonesia and access was granted on 25 November 2025. Informed consent was not sought because the dataset was secondary and anonymized and individuals could not be identified during or after data collection.

### Ethical approval

Ethical approval for this study was obtained from the Research Ethics Committee of the Faculty of Medicine, Universitas Sebelas Maret (Reference Number: 103/UN27.06.11/KEP/EC/2025). The ethics committee approved the study and waived the requirement for informed consent due to the use of secondary anonymized data.

### Data collection

All laboratory-confirmed malaria cases reported in Sorong City during the study period were included. A malaria case was defined as an infection confirmed by either microscopy or rapid diagnostic test (RDT) or both. Cases were classified by mode of detection based on the case-detection field recorded in SISMAL. Passive case detection (PCD) refers to facility-based presentation whereas active case detection (ACD) was performed during CHC outreach or home-visit screening. Microscopy was typically performed when patients presented to healthcare facilities (PCD), whereas RDTs were commonly administered during home visits by CHC staff or cadres (ACD). Because operational constraints (e.g., electricity outages or after-hours service) are not captured in SISMAL, the specific reason for selecting microscopy versus RDT could not be determined for each case. A standardized data extraction form was developed to retrieve patients’ information from SISMAL, including demographic data (age, gender, ethnicity, occupation, residential address, and pregnancy status), clinical data (methods of case detection and disease severity), laboratory data (diagnostic tests, Plasmodium species, and parasite load), treatment, and hospitalization status.

The endemicity level is determined by API; i.e., a number of confirmed malaria cases per 1,000 people at risk within one year [17]. API was classified as low (API < 1), moderate (API 1–5), or high (API > 5). Endemicity level for each patient was assigned using the API-based endemicity category recorded for the patient’s residential sub-area in SISMAL, which is maintained by the local malaria program based on routine surveillance and population-at-risk estimates. Parasite load was categorized in four levels, including low (<1,000 parasites/μl blood), moderate (1,000–4,999 parasites/μl blood), high (5,000–99,999 parasites/μl blood), and hyperparasitemia (≥100,000 parasites/μl blood) [18]. Parasite density and several clinical covariates are non-mandatory fields in SISMAL which has caused parasite load and clinical symptoms were incompletely recorded. Furthermore, concurrent diseases, past medical history (including prior malaria infections), and treatment outcomes were not reported. Those variables with incomplete and unavailable data were not used as primary predictors in regression analyses.

Severe malaria was defined as malaria in a patient presenting with at least one severe clinical manifestation, including shock, acute kidney failure, pulmonary oedema, impaired consciousness, jaundice, anemia, acidosis, hyperparasitemia (>10% of red blood cells parasitized with *P. falciparum* or parasite density >100.000/ul for *P. knowlesi*), prostration, convulsion, hypoglycemia or bleeding, while uncomplicated malaria was defined as malaria in a patient who has symptoms consistent with malaria and a positive parasitological test (microscopy or RDT) but without any feature of severe malaria [19,20].

Treatment practices were categorized as standard or non-standard according to the Indonesian national malaria treatment guideline [11]. “Inappropriate drug” referred to regimens that did not match the recommended drug combination for the recorded Plasmodium species, patient pregnancy status, and disease severity. “Inappropriate dose” referred to a recorded tablet number that did not match the national guideline-recommended dosing based on age and weight recorded in SISMAL.

Healthcare attendance was defined as the utilization of health facilities by patients when they perceive a health problem. For the purposes of this study, we compare the characteristics of malaria patients attending three different types of health facilities available in Sorong City (clinics, CHCs, and hospitals), and we examined predictors of healthcare attendance among all cases to identify access barriers, referral patterns, and case-management practices that affect resource use and quality of care. Among those presented to the hospitals, predictors for hospitalization were further analyzed.

### Statistical analysis

Data were incorporated into a Microsoft Excel spreadsheet and analyzed using IBM Statistical Package for Social Sciences (SPSS) version 26. Descriptive statistics, including mean ± standard deviation (SD) for continuous variables and frequency and percentage for categorical variables were used to summarize data. The distribution of categorical variables was examined, and where appropriate, categories with small cell counts were combined to ensure stable estimation while preserving conceptual meaning. Group comparisons were performed using independent t-test and one-way ANOVA for continuous variables and chi-square test for categorical variables. Since CHCs perform both passive and active case detection, only PCD cases were considered as individuals who actively perform “treatment-seeking” and present to healthcare facilities. Predictors of hospital attendance among all cases and predictors of hospitalization among those attending hospitals were examined using multiple logistic regression analysis. Based on the purposeful selection process, covariates for the regression model were identified and included variables that have a *p* value <0.25 in the univariate analysis as well as those that are clinically important based on the authors’ judgement (21, 22). In contrast, variables that had many missing values or with a low frequency were excluded from the regression model [21,22]. Variables included in the regression model for predictors of hospital attendance and hospitalization were age, gender, ethnicity, occupation, endemicity levels, pregnancy, and Plasmodium species. Age was entered as a categorical variable (<15 years vs ≥ 15 years) in the final multivariable models. Regression analyses used complete-case data for included covariates; variables with substantial missingness (e.g., parasite density) were summarized descriptively and were not included as primary predictors. A p-value of <0.05 was considered statistically significant.

## Results

### Demographic characteristics of patients

A total of 3,953 laboratory-confirmed malaria cases were identified in SISMAL during the study period, including one follow-up case. Most cases were reported by CHCs (n = 2,438; 61.7%), followed by hospitals (n = 1,011; 25.6%) and clinics (n = 504; 12.7%). Because CHCs report cases detected through both PCD (facility presentation) and ACD (outreach screening), CHC notifications reflect both detection pathways.

Among the 3,953 patients, most were aged ≥15 years old (n = 2,607; 65.9%), males (n = 2,349; 59.4%), of Papuan ethnicity (n = 2,142; 54.2%), and students (n = 1,446; 36.6%). The majority of patients lived in highly endemic areas (n = 3,084; 78%). Uncomplicated and severe malaria accounted for 99.9% (n = 3,951) and 0.1% (n = 2), respectively. There were statistically significant differences in the demographic characteristics and disease severity of malaria patients attending clinics, CHCs, and hospitals (p = 0.001 and 0.003) (**Table 1**).

**Table 1:** Demographic profile and disease severity of 3,953 malaria cases recorded in SISMAL across five clinics, 10 community health centers (CHCs), and seven hospitals in Sorong City from 1 January to 31 December 2024.

Characteristics*	Total (n = 3,953)	Clinics (n = 504; 12.7%)	CHCs (n = 2,438; 61.7%)	Hospitals (n = 1,011; 25.6%)	*P* value
**Age**	23.6 ± 16.3 [1–88]	23.6 ± 16.4 [1–71]	21.6 ± 15.6 [1–88]	28.6 ± 16.9 [1–81]	**0.001** ^**a**^
**Age group**					**0.001** ^**b**^
≥15 years	2,607 (65.9)	341 (67.7)	1,464 (60)	802 (79.3)	
<15 years	1,346 (34.1)	163 (32.3)	974 (40)	209 (20.7)	
**Gender**					**0.001** ^**b**^
Male	2,349 (59.4)	317 (62.9)	1,392 (57.1)	640 (63.3)	
Female	1,604 (40.6)	187 (37.1)	1,046 (42.9)	371 (36.7)	
**Ethnicity**					**0.001** ^**b**^
Papuan	2,142 (54.2)	306 (60.7)	1,394 (57.2)	442 (43.7)	
Non-Papuan	1,811 (45.8)	198 (39.3)	1,044 (42.8)	569 (56.3)	
**Occupation**					**0.001** ^**b**^
Employed					
Outdoor^α^	192 (4.9)	62 (12.3)	89 (3.6)	41 (4.1)	
Indoor^β^	229 (5.8)	18 (3.6)	79 (3.2)	132 (13.1)	
Others^γ^	346 (8.7)	18 (3.6)	183 (7.5)	145 (14.3)	
Housewife	484 (12.2)	67 (13.3)	321 (13.2)	96 (9.5)	
Student	1,446 (36.6)	174 (34.5)	1,064 (43.6)	208 (20.6)	
Unemployed	1,256 (31.8)	165 (32.7)	702 (28.8)	389 (38.5)	
Endemicity levels of residential address**					**0.001** ^**b**^
High (API > 5)	3,084 (78)	342 (67.8)	2,137 (87.7)	605 (59.8)	
Moderate (API 1–5)	808 (20.4)	141 (28)	293 (12)	374 (37)	
Low (API < 1)	61 (1.5)	21 (4.2)	8 (0.3)	32 (3.2)	
Pregnancy (n = 1,604)***					**0.003** ^**b**^
Yes	27 (1.7)	0 (0)	26 (2.5)	1 (0.03)	
No	1,577 (98.3)	187 (100)	1,020 (97.5)	370 (99.7)	
**Disease severity**					**0.001** ^**b**^
Uncomplicated	3,951 (99.9)	502 (99.6)	2,438 (100)	1,011 (100)	
Severe	2 (0.1)	2 (0.4)^&^	0 (0)	0 (0)	

* Continuous variables are presented as mean ± SD [Min – Max], categorical variables are presented as n (%)

** Based on annual parasite incidence (API) associated with the patient’s residential address recorded in SISMAL

*** Analysis on female patients only

a One-way ANOVA

b Chi-square

α Farmer, gardener, fisherman, soldier, miner, forest worker

β Office worker

γ Laborer, retailer, policeman, unspecified employment

& The first patient was a 22-year-old non-Papuan male, had *P. vivax* infection detected by RDT, no information regarding clinical symptoms and referral. The second patient was a 23-year-old non-Papuan male, had *P. vivax* infection detected by RDT, he was referred to hospital due to thrombocytopenia.

### Diagnosis and treatment of patients

Microscopy was the most commonly used diagnostic method across all healthcare facilities (n = 2,772; 70.1%, p = 0.001). Parasite density was recorded for 799 of 3,953 cases (20.2%), with the highest completeness among clinics. Among those with available parasite density data, CHC patients predominantly had low-to-moderate parasitemia, while high parasitemia was more common among patients attending clinics and hospitals (p = 0.001) (**Table 2**). Given the substantial missingness, parasite-density comparisons should be interpreted cautiously. Furthermore, since parasite load was only available in 3.4% (83/2,438) of CHC patients, a formal statistical comparison between ACD and PCD subgroups was deemed inappropriate.

**Table 2:** Diagnosis and treatment of 3,953 malaria cases recorded in SISMAL across five clinics, 10 community health centers (CHCs), and seven hospitals in Sorong City from 1 January to 31 December 2024.

Characteristics*	Total (N = 3,953)	Clinics (N = 504; 12.7%)	CHCs (N = 2,438; 61.7%)	Hospitals (N = 1,011; 25.6%)	*P* value^#^
**Confirmatory diagnosis**					**0.001**
Microscopy	2,772 (70.1)	440 (87.3)	1,400 (57.4)	932 (92.2)	
RDT	928 (23.5)	32 (6.3)	828 (34)	68 (6.7)	
Microscopy and RDT	253 (6.4)	32 (6.3)	210 (8.6)	11 (1.1)	
**Parasite load (N = 799)**	6,816.91 ± 14,711.96^α^	5,966.23 ± 3,951.29^β^	3,071.84 ± 4,332^γ^	9,518.07 ± 24,955.6^δ^	**0.001**
Parasite load group (among those with available data)**					**0.001**
Low	133 (16.6)	31 (6.78)	34 (41)	68 (26.2)	
Moderate	280 (35)	181 (39.6)	35 (42.1)	64 (24.7)	
High	382 (47.8)	245 (53.6)	14 (16.9)	123 (47.5)	
Hyperparasitemia	4 (0.5)	0 (0)	0 (0)	4 (1.5)^ε^	
Malaria species infection and treatment***					
***P. falciparum***	**1,222 (30.9)**	**263 (52.2)**	**720 (29.5)**	**239 (23.6)**	**0.001**
**Standard** ^**a**^	**980 (80.2)**	**233 (88.6)**	**581 (80.7)**	**166 (69.5)**	**0.001**
**Non-standard**	**242 (19.8)**	**30 (11.4)**	**139 (19.3)**	**73 (30.5)**	
Inappropriate drug	101 (41.7)	12 (40)	80 (57.6)	9 (12.3)	
Inappropriate dose	112 (46.3)	17 (56.7)	38 (27.3)^&^	57 (78.1)	
Not treated	29 (12)	1 (3.3)	21 (2.9)	7 (9.6)	
***P. vivax***	**2,588 (65.5)**	**232 (46)**	**1,594 (65.4)** ^&&^	**762 (75.4)**	**0.001**
**Standard** ^**b**^	**2,032 (78.5)**	**189 (81.5)**	**1,293 (81.1)**	**550 (72.2)**	**0.001**
**Non-standard**	**556 (21.5)**	**43 (18.5)**	**301 (18.9)**	**212 (27.8)**	
Inappropriate drug	234 (42.1)	16 (37.2)	208 (69.1)	10 (4.7)	
Inappropriate dose	261 (46.9)	23 (53.4)	50 (16.6)^&^	188 (88.7)	
Not treated	61 (11)	4 (9.3)	43 (14.3)	14 (1.8)	
***P. ovale*** ^**b**^	**2 (0.05)**	**0 (0)**	**1 (0.04)**	**1 (0.1)**	–
**Non-standard**	**2 (100)**	–	**1 (100)**	**1 (100)**	
Inappropriate drug	2 (100)	–	1 (100)	1 (100)	
***P. knowlesi* (probable)** ^**c**^	**1 (0.02)**	**0 (0)**	**0 (0)**	**1 (0.1)**	–
**Not standard**	**1 (100)**	–	–	**1 (100)**	
Inappropriate drug	1 (100)	**–**	**–**	1 (100)	
**Mixed-species infections**	**140 (3.5)**	**9 (1.8)**	**123 (5)**	**8 (0.8)**	**0.015**
**Standard** ^**b**^	**116 (82.9)**	**9 (100)**	**102 (82.9)**	**5 (62.5)**	**0.025**
**Non-standard**	**24 (17.1)**	**0 (0)**	**21 (17.1)**	**3 (37.5)**	
Inappropriate drug	13 (54.2)	0 (0)	12 (57.1)	1 (33.3)	
Inappropriate dose	7 (29.2)	0 (0)	5 (23.8)	2 (66.7)	
Not treated	4 (16.7)	0 (0)	4 (19)	0 (0)	

* Continuous variables are presented as Mean ± SD [Min – Max], categorical variables are presented as n (%)

** Low (< 1000 parasites/μL blood), moderate (1,000–4,999 parasites/μL blood), high (5,000–99,999 parasites/μL blood), hyperparasitemia (≥100,000/μL blood). Completing data on parasite load is not mandatory in SISMAL, only 20.2% (799/3,953) of cases documented parasite load.

*** Standard treatment relies on national guidelines and is assessed based on types of drugs and dosage determined by patients’ age, weight, pregnancy status, Plasmodium species, and disease severity. Primaquine is contraindicated in pregnant women or infants under 6 months. Severe malaria is treated with intravenous artesunate 2.4 mg/kg in 0, 12, and 24 hours, followed by 2.4 mg/kg/day until the patient can take oral medication.

α Available in 799 of 3,953 total cases (20.2%)

β Available in 457 of 504 cases (90.7%) presenting to clinics

γ Available in 83 of 2,438 cases (3.4%) presenting to CHCs

δ Available in 259 of 1,011 (25.6%) cases presenting to hospitals

ε All patients had *P. falciparum* and were reported as “uncomplicated malaria” in SISMAL

& Including 2 pregnant women receiving a combination of DHP and primaquine, both are not anemic

&& Including 1 case with diagnosis “*P. vivax*, probable knowlesi”, examined by microscopy

a Uncomplicated *P. falciparum* is treated with DHP plus a single 0.25 mg/kg primaquine dose (as a gametocytocide).

b Uncomplicated *P. vivax*, *P. ovale*, and mix *P. falciparum + P. vivax/P. ovale* receives DHP plus primaquine 0.25 mg/kg daily for 14 days for radical cure. One patient with malaria vivax received quinine; no information exists whether it is used as the second-line drug (for those with DHP failure) or due to drug stock-outs.

c Standard treatment for *P. knowlesi* is DHP, without primaquine

# Chi-square

There was a statistically significant difference in the distribution of Plasmodium species across healthcare facilities (p = 0.001). Regarding species distribution, most cases were *P. vivax* (n = 2,588; 65.5%), followed by *P. falciparum* (n = 1,222; 30.9%) which accounted for approximately half of clinic cases (263/504; 52.2%). Two patients were infected with *P. ovale*, and both were treated with DHP monotherapy. A single case was identified by microscopy as “probable knowlesi*”* in a 37-year-old Papuan male (parasite density: 3,636 parasites/µL). Another case was reported as “*P. vivax,* probable knowlesi*”* infection in an 8-year-old Papuan student. However, this remains an unconfirmed morphological diagnosis. Both cases were treated as outpatients with a 3-day course of DHP plus 14 days of primaquine.

The combination of DHP and primaquine was the most common treatment regimen, administered to almost 90% of patients with *P. falciparum*, *P. vivax*, and mixed infections. Primaquine monotherapy was given to 5.4% (66/1,222) of *P. falciparum*, 6.4% (166/2,588) of *P. vivax*, and 7.9% (11/140) of mixed infection cases. In contrast, 5.6% (144/2,588) of *P. vivax* patients did not receive primaquine (data not shown). Overall, 79.1% (3,128/3,953) of cases received standard treatment according to national malaria guidelines. These estimates reflect the appropriateness of treatments based on surveillance data documented in SISMAL.

### Predictors of hospital attendance

Several demographic, occupational, and epidemiological factors were significantly associated with healthcare attendance (**Table 3**). There was a total of 3,208 PCD cases reported by CHCs, clinics, and hospitals. Predictors for hospital attendance include older age (aOR: 1.02; 95% CI: 1.00–1.02; p = 0.001), non-Papuan ethnicity (aOR: 1.77; 95%CI: 1.51–2.07; p = 0.001), being housewife (aOR: 1.57; 95%CI: 1.02–2.42; p = 0.041) or student (aOR: 1.56; 95%CI: 1.05–2.31; p = 0.029), and living in moderate- (aOR: 2.01; 95%CI: 1.69–2.40; p = 0.001) or low- (aOR: 4.57; 95%CI: 2.01–10.39; p = 0.001) endemicity areas.

**Table 3:** Predictors of hospital attendance among 3,208 passively detected malaria cases presenting to healthcare facilities in Sorong City from 1 January to 31 December 2024.

Characteristics*	Univariable analysis				Multiple logistic regression analysis	
**Non-hospitals^ (N = 2,197)**	**Hospitals (N = 1,011)**	***t*/ OR (95% CI)**	***P* value**	**Adjusted OR (95% CI)**	***P* value**
**Age**	23.18 ± 15.24 [1–88]	28.63 ± 16.87 [1–81]	−8.76	**0.001** ^**a**^	1.02 (1.00–1.02)	**0.001**
≥15 years	1,466 (66.7)	802 (79.3)	1.91 (1.60-2.28)	**0.001** ^**b**^	1.18 (0.94–1.48)	0.165
<15 years^#^	731 (33.3)	209 (20.7)	–	–	–	–
**Gender**						
Male^#^	1,318 (60)	640 (63.3)	–	–	–	–
Female	879 (40)	371 (36.7)	0.87 (0.75-1.01)	0.074^b^	0.96 (0.82–1.13)	0.615
**Ethnicity**						
Papuan^#^	1,325 (60.3)	442 (43.7)	–	–	–	–
Non-Papuan	872 (39.7)	569 (56.3)	1.96 (1.68-2.28)	**0.001** ^**b**^	1.77 (1.51–2.07)	**0.001**
**Occupation**						
Employed						
Outdoor^α#^	124 (5.6)	38 (3.8)	–	–	–	–
Indoor^β^	123 (5.6)	62 (6.1)	1.65 (1.02–2.64)	0.040^b^	1.62 (0.99–2.65)	0.054
Others^γ^	194 (8.8)	92 (9.1)	1.55 (0.99–2.40)	0.052^b^	1.61 (1.02–2.54)	0.041
Housewife	272 (12.4)	133 (13.2)	1.60 (1.05–2.43)	**0.029** ^**b**^	1.57 (1.02–2.42)	**0.041**
Student	777 (35.4)	372 (36.8)	1.56 (1.06–2.29)	**0.023** ^**b**^	1.56 (1.05–2.31)	**0.029**
Unemployed	707 (32.2)	314 (31.1)	1.45 (0.98–2.13)	0.060^b^	1.48 (0.99–2.21)	0.056
Endemicity levels of residential address**						
High (API > 5) ^#^	1,777 (80.9)	659 (65.2)	–	–	–	–
Moderate (API 1–5)	410 (18.7)	337 (33.3)	2.22 (1.87–2.63)	**0.001** ^**b**^	2.01 (1.69–2.40)	**0.001**
Low (API < 1)	10 (0.5)	15 (1.5)	4.05 (1.80–9.05)	**0.001** ^**b**^	4.57 (2.01–10.39)	**0.001**
**Plasmodium species**						
*P. falciparum* ^#^	669 (30.5)	315 (31.2)	–	–	–	–
*P. vivax*	1,447 (65.9)	652 (64.5)	0.96 (0.81–1.13)	0.596^b^	0.95 (0.80–1.12)	0.520
*P. ovale*	1 (0.05)	1 (0.1)	2.12 (0.13–34.06)	0.595^**b**^	1.71 (0.11–27.57)	0.707
*P. knowlesi* (probable)	1 (0.05)	0 (0)	0.00 (0.00 - ~)	1.00^**b**^	0.00	1.00
Mixed infections	79 (3.6)	43 (4.3)	1.16 (0.78–1.72)	0.472^b^	1.14 (0.75–1.71)	0.543

* Continuous variables are presented as Mean ± SD [Min – Max], categorical variables are presented as n (%)

** Based on annual parasite incidence (API) associated with the patient’s residential address recorded in SISMAL

*** Analysis was performed for female patients only

^ Non-hospitals include community health centers and clinics

# Reference

α Including farmer, gardener, fisherman, soldier, miner, forest worker

β Office worker

γ Laborer, retailer, policeman, unspecified employment

a Independent t-test

b Chi-square test

NA: not available

Occupation also influenced hospital attendance. Office workers (aOR 5.2; 95% CI 3.21–8.44; p = 0.001) and unemployed individuals (aOR 3.3; 95% CI 2.15–5.06; p = 0.001) were more likely to attend hospitals. Endemicity levels of residence were also associated with facility choice: individuals living in moderate (aOR 2.84; 95% CI 2.38–3.4; p = 0.001) or low (aOR 6.08; 95% CI 3.54–10.44; p = 0.001) endemicity areas were more likely to seek care at hospitals. Those who were not pregnant were also more likely to visit hospitals (aOR 8.14; 95% CI 1.07–61.72; p = 0.043).

### Predictors of hospitalization

Among patients who presented to hospitals, age < 15 years was the only significant predictor of hospitalization (aOR 1.9; 95% CI 1.18–3.06; p = 0.009) (Table 4). No other factors were significantly associated with inpatient admission.

**Table 4:** Predictors of hospitalization among 1,011 patients presented to hospitals in Sorong City from 1 January to 31 December 2024.

Variables*	Univariable analysis				Multiple logistic regression analysis	
**Outpatients (N = 250)**	**Inpatients (N = 761)**	***t*/ OR (95% CI)**	***P* value**	**Adjusted OR (95% CI)**	***P* value**
**Age (years)**	29 ± 16.3 [1–75]	28 ± 17.1 [1–81]	−0.46	0.647^a^	1.00 (0.99–1.02)	0.657
**Age group**						
≥15 years^#^	210 (84)	592 (77.8)	–	–	–	–
<15 years	40 (16)	169 (22.2)	1.50 (1.03-2.19)	**0.036** ^**b**^	1.90 (1.18–3.06)	**0.009**
**Gender**						
Male^#^	162 (64.8)	478 (62.8)	–	–	–	–
Female	88 (35.2)	283 (37.2)	1.09 (0.81-1.47)	0.572^b^	1.18 (0.84–1.66)	0.349
**Ethnicity**						
Papuan^#^	111 (44.4)	331 (43.5)	–	–	–	–
Non-Papuan	139 (55.6)	430 (56.5)	1.04 (0.78-1.38)	0.802^b^	0.98 (0.72–1.33)	0.902
**Occupations**						
Employed						
Outdoor^α#^	8 (0)	33 (0.1)	–	–	–	–
Indoor^β^	23 (2)	109 (0.9)	1.09 (0.40–2.96)	0.860^b^	1.19 (0.43–3.30)	0.732
Others^γ^	32 (3.9)	113 (12.5)	0.814 (0.31–2.14)	0.677^b^	0.89 (0.33–2.42)	0.818
Housewife	26 (10.4)	70 (9.2)	0.62 (0.23–1.68)	0.349^b^	0.57 (0.20–1.61)	0.285
Student	48 (19.2)	160 (21)	0.77 (0.30–1.98)	0.586^b^	0.68 (0.25–1.86)	0.451
Unemployed	113 (45.2)	276 (36.3)	0.56 (0.23–1.41)	0.219^b^	0.50 (0.19–1.29)	0.149
Endemicity levels of residential address**						
High API	150 (60)	455 (59.8)	–	–	–	–
Moderate API	95 (38)	279 (36.7)	0.97 (0.72–1.30)	0.831^b^	0.86 (0.62–1.18)	0.349
Low API	5 (2)	26 (3.4)	1.78 (0.67–4.71)	0.245^b^	1.71 (0.64–4.57)	0.287
**Pregnancy status (n = 371)**						
Yes^#^	0 (0)	1 (0.4)	–	–	–	–
No	88 (100)	282 (99.6)	0.00	1.00^b^	0.00	1.00
**Plasmodium species**						
*P. falciparum* ^#^	59 (23.6)	180 (23.6)	–	–	–	–
*P. vivax*	188 (75.2)	575 (75.6)	1.00 (0.71–1.40)	0.996^b^	1.02 (0.72–1.45)	0.911
*P. ovale*	0 (0)	1 (0.1)	NA	1.00^b^	NA	1.00
*P. knowlesi* (probable)	1 (0.4)	0 (0)	NA	1.00^b^	NA	1.00
Mixed infections	2 (0.8)	5 (0.7)	0.98 (0.19–5.00)	0.984^b^	1.03 (0.20–5.42)	0.971

* Continuous variables are presented as Mean ± SD [Min – Max], while categorical variables are presented as n (%)

** Based on annual parasite incidence (API) associated with the patient’s residential address recorded in SISMAL

a Independent t-test

b Chi-square test

# reference group in logistic regression

α Including farmer, gardener, fisherman, soldier; ^β^ Office worker; ^γ^ retailer, policeman, and unspecified employment

NA: not available

## Discussion

Malaria remains a major cause of morbidity and mortality, particularly among vulnerable groups such as young children, pregnant women, and populations living in disadvantaged settings. Papua continues to experience persistent transmission, reflecting systemic challenges in malaria control [23]. In Sorong City, the API in 2024 reached 13.23 per 1,000 population at risk, far above Indonesia’s elimination target of <1 per 1,000, underscoring the severity of the malaria burden in this area [3].

This study found that most malaria cases occurred among individuals aged ≥15 years, with males slightly predominating, consistent with findings from Papua and Papua New Guinea, where both genders experience similar exposure patterns in high-transmission zones [24,25]. Students and unemployed individuals comprised a substantial proportion of recorded cases; however, this study did not assess occupation-specific malaria risk, and these patterns may reflect the underlying population structure and detection pathways. Although prior studies suggest that outdoor work, geographic access, and socioeconomic factors can influence malaria risk and healthcare use [26–29], these variables (e.g., travel time/distance, education, household income) were not reported in SISMAL and therefore cannot be evaluated as drivers in this dataset. Nevertheless, the high case burden recorded at CHCs supports strengthening community-based services and targeted engagement with groups frequently represented in notifications (e.g., students and those unemployed), alongside locally appropriate outreach to reduce barriers to timely diagnosis and treatment.

Microscopy remained the primary diagnostic tool in Sorong City so that the quality of malaria laboratory services is essential for establishing a diagnosis and depends heavily on the competence and performance of laboratory personnel at every level of health care facilities. The quality of examinations is monitored through cross-checking mechanisms at the district/city, provincial, and national levels and the competency of laboratory personnels is maintained through workshops and external assessment [5]. However, variations in diagnostic capacity across facilities and the absence of routinely documented external quality assurance in SISMAL may affect species attribution. In addition, “classification errors” in this context may reflect electronic reporting and/or data-entry issues rather than laboratory microscopy performance [30]. The dominance of *P. vivax* aligns with regional trends [31,32] and is programmatically important because effective control requires not only treatment of blood-stage infection but also high-quality radical cure and adherence support to prevent relapse. Although most patients received DHP plus primaquine as recommended [33], treatment gaps such as primaquine or DHP monotherapy indicate non-standard regimens recorded in surveillance data; the underlying causes (e.g., incomplete documentation, provider practice variation, or drug availability constraints) could not be distinguished in this study and should be interpreted cautiously [34]. Strengthening routine guideline training, supervision, and linkage between case data and commodity/stock monitoring could help reduce non-standard regimens.

Misdiagnosis risk remains a major challenge in regions where multiple Plasmodium species circulate. Light microscopy can fail to differentiate species because of morphological overlap [35], and misclassification has been widely reported [36,37]. While one case in this dataset was recorded as “probable knowlesi*”* and another case was recorded as “*P. vivax,* probable knowlesi*”* infection via microscopy*,* we emphasize that these were not molecularly confirmed. Given the morphological similarities between *P. knowlesi* and other Plasmodium species, this finding should be interpreted cautiously [38]. Also, given the very small number observed, we do not draw inferences about zoonotic transmission patterns in Sorong City from these data. Where zoonotic malaria is suspected, expanding diagnostic capacity using appropriate RDTs and/or molecular tools may improve ‌‌diagnostic certainty [38].

It has been reported that in 2024, the Southwest Papua province achieved 82.34% of malaria standard treatment [3]. Non-standard treatment records such as primaquine monotherapy or missing primaquine documentation warrant careful attention. Primaquine is not indicated as monotherapy for acute blood-stage malaria. For *P. falciparum,* it is administered as a single-dose gametocytocidal agent alongside ACT to reduce transmission. For *P. vivax,* it remains the primary agent for radical cure [39]. However, its programmatic success is often limited by poor adherence to the standard 14-day regimen and the risk of hemolysis in G6PD-deficient individuals, necessitating supervised administration or simplified high-dose regimens where feasible. Because this study used routine surveillance data where age and body weight were not verified, dose assessment should be interpreted as an approximation and may be influenced by data-entry error. In addition, clinical context was incompletely recorded, we cannot determine whether non-standard regimens reflect true clinical practice, justified clinical contraindications, incomplete documentation, or data-entry error. Untreated cases should be interpreted with caution as this may reflect missing treatment entries, incomplete recording in surveillance data, or drug stockouts. Nonetheless, routine treatment audits, supportive supervision, and strengthened recording of contraindications and follow-up plans for radical cure could help reduce inappropriate case management [40]. It is also important to note that standard treatment relies on drug availability, so good drug management is needed to ensure that there is no stock-out of malaria drugs.

Predictors of hospital attendance included older age, non-Papuan ethnicity, being housewife or student, and residence in low- or moderate-endemic areas. These associations should be interpreted cautiously because key determinants such as socioeconomic status, education, and geographic access were not measured in SISMAL, and markers of clinical severity were rare (only 2 severe malaria cases) and incompletely recorded (parasite density available for ~20%). Therefore, while the observed pattern is consistent with non-clinical factors influencing facility attendance, this study cannot directly attribute hospital attendance to socioeconomic or geographic drivers [41]. Among those who reached hospitals, only age < 15 years predicted hospitalization. Given that most cases were recorded as uncomplicated while hospitalization was common, additional unmeasured factors (e.g., local admission practices, clinician risk perception, comorbidities, referral protocols, or documentation practices) may contribute and should be explored in future work.

The data quality challenges identified in this study, such as gaps in non-mandatory fields and reporting inconsistencies, may be common limitations of routine surveillance systems [42]. Nevertheless, these findings provide a critical evidence base for health authorities to refine the SISMAL system. These findings suggest that moving toward malaria elimination in Sorong City will require a shift toward evidence-driven supervision. Specifically, our results can inform the development of clearer data entry standards and the implementation of periodic quality audits. By identifying these systemic gaps, this study provides the necessary justification for targeted training of facility-level malaria staff, ensuring that routine data can reliably guide future elimination strategies.

This study has several limitations related to the study setting and study period. The data was confined to Sorong City, limiting generalizability to other malaria endemic regions with differing vector ecologies and healthcare capacities. Furthermore, temporal trends and year-to-year variability could not be evaluated since the analysis was based on a single year of surveillance data.

A key limitation of this study is that surveillance data depend heavily on completeness and accuracy, restricting analysis of treatment adequacy and disease severity due to missing parasitemia or clinical variables. Missing information about prior malaria infection and its treatment, current clinical symptoms and laboratory results (e.g., parasitemia density, hemoglobin levels) restricted the evaluation of treatment appropriateness as well as disease severity and its association with demographic profile and laboratory findings. Although we did not analyze the clinical symptoms, active screening led by CHCs likely facilitated the detection of malaria among asymptomatic individuals. In endemic areas like Sorong City, partial immunity may allow individuals to remain asymptomatic while harboring parasites. These individuals serve as persistent reservoirs because they can still carry gametocytes capable of infecting mosquito vectors. Our findings suggest that malaria elimination in Papua cannot rely exclusively on hospital-based passive detection; instead, targeted active screening in transmission “hotspots” is essential to identify and clear asymptomatic reservoirs and interrupt local transmission. Misclassification of mixed or zoonotic infections likely occurred given reliance on microscopy without molecular confirmation [43] and reporting delays in SISMAL may contribute to discrepancies between medical records and surveillance data.

Last but not least, behavioral drivers of care seeking among passively detected cases could not be directly measured and warrant future qualitative inquiry. This study relies on national surveillance data, which lacks granular socioeconomic and cultural variables, such as education level, income, and distance to facilities, that are known to influence healthcare utilization. Consequently, while our quantitative analysis identifies broad patterns, further qualitative research is required to provide a deeper understanding of the barriers to treatment seeking in this region.

Future research should integrate molecular diagnostics, expand surveillance across Papua, and link SISMAL with medical records using unique identifiers to improve completeness of clinical variables and treatment documentation. Mixed-methods approaches (including qualitative work) could clarify why patients attend hospitals versus primary facilities and why hospitalization is frequent despite predominantly uncomplicated malaria classifications. Considering the lack of data in the national surveillance system, better management and auditing is urgently required to produce complete and reliable data. Strengthening digital infrastructure and promoting interoperable data governance will be essential to advancing Indonesia’s malaria elimination goals.

## Conclusions

Malaria remains a major burden in Sorong, driven by *P. vivax*. Strengthening surveillance, diagnostics, and equitable access is essential. Improving adherence to national case-management guidelines, particularly documentation and delivery of effective *P. vivax* radical cure where safe and appropriate, and strengthening primary-care capacity may support timely diagnosis, reduce avoidable hospital attendance and admissions, and advance Indonesia’s 2030 elimination goals in endemic communities.
